# Association between tobacco company corporate social responsibility claims, support for the company and policies, and perceptions and intentions among Brazilians

**DOI:** 10.1590/0102-311XEN111925

**Published:** 2026-02-13

**Authors:** Luciana Correia Borges, Silvana Rubano Barretto Turci, Sara Perez, Eric Crosbie, Louis Forline, Graziele Grilo, Jennifer L. Pearson

**Affiliations:** 1 University of Nevada, Reno, U.S.A.; 2 Fundação Oswaldo Cruz, Rio de Janeiro, Brasil.; 3 Institute for Global Tobacco Control, Johns Hopkins University, Baltimore, U.S.A.

**Keywords:** Tobacco Industry, Social Marketing, Public Policy, Public Opinion, Indústria do Tabaco, Marketing Social, Política Pública, Opinião Pública, Industria del Tabaco, Mercadeo Social, Política Pública, Opinión Pública

## Abstract

This study aimed to examine how corporate social responsibility messaging from tobacco companies influences public attitudes, policy support, and risk perceptions among Brazilian adults. Specifically, it assessed whether exposure to a fictional tobacco company’s corporate social responsibility video affects viewers’ opinions of the company, perceptions of cigarette and e-cigarette harm, and intentions to use these products. An online randomized experiment was conducted with 4,047 Brazilian adults aged 18 and older, recruited via the Qualtrics panel. Participants were randomly assigned to view a three-minute video depicting a fictitious Brazilian tobacco company, Cruzeiro do Sul, either presenting its business activities alone (control) or with additional corporate social responsibility messaging (experimental). Outcomes were assessed using linear regression for continuous variables and Poisson regression with robust variance for binary outcomes. Interaction effects were examined by gender and smoking status. Exposure to the corporate social responsibility video was significantly associated with more favorable views of the company (β = 0.16, p < 0.001), especially among women (β = 0.21). Viewers were also more likely to perceive the company’s cigarettes as less harmful than others (IRR = 1.14, 95%CI: 1.05; 1.25), with a stronger effect among nonsmokers (IRR = 1.26, 95%CI: 1.04; 1.53). No significant effect was observed on overall support for tobacco control policies or intentions to use the products. Corporate social responsibility messaging by tobacco companies can improve corporate image and mislead consumers about product harm, potentially undermining tobacco control efforts. These effects mirror those observed with traditional tobacco advertising.

## Abstract (pt)

Objetivou-se examinar como as mensagens de responsabilidade social corporativa das empresas de tabaco influenciam as atitudes públicas, o apoio político e a percepção de risco entre os adultos brasileiros; especificamente, explorar se a exposição ao vídeo de responsabilidade social corporativa de uma empresa fictícia de tabaco afeta as opiniões dos espectadores sobre a empresa, as percepções dos danos do cigarro e do cigarro eletrônico e a intenção de usar esses produtos. Realizou-se um experimento randomizado online com 4.047 adultos brasileiros com 18 anos ou mais, recrutados por meio do painel Qualtrics. Os participantes foram aleatoriamente designados para assistir a um vídeo de três minutos retratando uma empresa fictícia de tabaco brasileira - a Cruzeiro do Sul -, apresentando suas atividades comerciais isoladas (controle) ou com mensagens adicionais de responsabilidade social corporativa (experimental). Os desfechos foram avaliados por meio de regressão linear para as variáveis contínuas e regressão de Poisson com variância robusta para os desfechos binários. Os efeitos de interação foram explorados por gênero e tabagismo. A exposição ao vídeo de responsabilidade social corporativa foi associada a opiniões significativamente mais favoráveis da empresa (β = 0,16, p < 0,001), especialmente entre as mulheres (β = 0,21). Os espectadores também eram mais propensos a perceber os cigarros da empresa como menos prejudiciais do que outros (IRR = 1,14, IC95%: 1,05; 1,25), com um efeito mais forte entre os não fumantes (IRR = 1,26, IC95%: 1,04; 1,53). Não houve efeito significativo no apoio geral às políticas de controle do tabaco ou na intenção de usar os produtos. As mensagens de responsabilidade social corporativa das empresas de tabaco podem melhorar a imagem corporativa e enganar os consumidores sobre os danos causados pelo produto, potencialmente minando os esforços de controle do tabaco. Esses efeitos refletem aqueles observados na publicidade tradicional do tabaco.

## Abstract (es)

El objetivo era examinar cómo los mensajes de responsabilidad social corporativa de las empresas de tabaco influyen en las actitudes del público, el apoyo a las políticas y la percepción del riesgo entre los adultos brasileños. En concreto, se trataba de explorar si la exposición al vídeo de responsabilidad social corporativa de una empresa de tabaco ficticia afecta a la opinión de los espectadores sobre la empresa, a su percepción de los daños causados por los cigarrillos y los cigarrillos electrónicos, y a su intención de consumir estos productos. Se llevó a cabo un experimento aleatorio en línea con 4.047 adultos brasileños mayores de 18 años, reclutados a través del panel Qualtrics. Los participantes fueron asignados aleatoriamente para ver un vídeo de tres minutos que mostraba una empresa de tabaco brasileña ficticia, Cruzeiro do Sul, presentando solo sus actividades comerciales (control) o con mensajes adicionales de responsabilidad social corporativa (experimental). Los resultados se evaluaron utilizando regresión lineal para las variables continuas y regresión de Poisson con varianza robusta para los resultados binarios. Se exploraron los efectos de interacción por género y hábito tabáquico. La exposición al vídeo de responsabilidad social corporativa se asoció significativamente con opiniones más favorables sobre la empresa (β = 0,16, p < 0,001), especialmente entre las mujeres (β = 0,21). Los espectadores también eran más propensos a percibir los cigarrillos de la empresa como menos nocivos que otros (IRR = 1,14, IC95%: 1,05; 1,25), con un efecto más marcado entre los no fumadores (IRR = 1,26, IC95%: 1,04; 1,53). No se observó ningún efecto significativo en el apoyo general a las políticas de control del tabaco ni en la intención de consumir los productos. Los mensajes de responsabilidad social corporativa de las empresas de tabaco pueden mejorar la imagen corporativa y engañar a los consumidores sobre los daños del producto, lo que podría minar los esfuerzos de control del tabaco. Estos efectos son similares a los observados con la publicidad tradicional del tabaco.

## Introduction

Broadly defined, “corporate social responsibility” is the idea that corporations have an ethical obligation to improve society beyond generating profits and enriching shareholders ^1^. Some companies, such as Lego, even incorporate corporate social responsibility in their by-laws aimed at contributing to societal well-being ^2^. Companies may also engage in corporate social responsibility out of enlightened self-interest, recognizing that integrating corporate social responsibility into their overall strategy can yield competitive advantages by enhancing brand reputation, attracting customers, and fostering innovation ^2^. Corporate social responsibility initiatives are also used to reduce regulatory oversight by implementing practices that minimize the risk of negative attention from the public and regulators, or by otherwise managing a company’s public image ^2^ ^,^ ^3^ ^,^ ^4^.

While corporate social responsibility initiatives can benefit corporations and contribute to societal well-being, tobacco companies’ use of corporate social responsibility presents a stark conflict of interest ^5^. Their profit mandate, which is fundamentally at odds with public health goals of eliminating death and disease caused by their products, raises serious ethical concerns ^6^. Major tobacco companies such as Philip Morris International and British American Tobacco have employed corporate social responsibility to block, weaken, and delay tobacco control policies ^7^. Tobacco companies also use corporate social responsibility to counteract negative publicity, increase share value, and ensure long-term business sustainability, with no evidence of substantive changes in their core business practices ^8^ ^,^ ^9^ ^,^ ^10^ ^,^ ^11^ ^,^ ^12^ ^,^ ^13^. Also, these companies commonly employ corporate social responsibility to gain access to policymakers and cultivate allies ^8^ ^,^ ^9^ ^,^ ^10^ ^,^ ^11^ ^,^ ^12^ ^,^ ^13^. Furthermore, tobacco companies use corporate social responsibility claims to position their brands as socially responsible (e.g., corporate social responsibility-related eco-friendly language in cigarette advertisements) to differentiate their products from those of competitors ^14^.

Recent studies reveal a global consistency in the tobacco industry’s corporate social responsibility strategies, with comparable initiatives observed across various countries. These include high-income nations such as Greece and France, as well as low- and middle-income countries such as India and Brazil, suggesting that companies follow a common approach to their corporate social responsibility efforts ^12^ ^,^ ^15^ ^,^ ^16^. Brazil’s status as the world’s largest tobacco exporter and third-largest producer ^17^, coupled with its history of progressive tobacco control policies ^18^, makes it an ideal setting for such analysis, particularly given tobacco companies’ strategic use of corporate social responsibility initiatives to oppose regulation.

Although Brazil has strict regulation banning tobacco advertising, promotion, and sponsorship (TAPS), it does not explicitly classify corporate social responsibility as a form of advertising or sponsorship under Article 13 of the World Health Organization (WHO) Framework Convention on Tobacco Control (FCTC). It also lacks specific regulations to control or ban the public promotion of corporate social responsibility activities, as recommended in the FCTC Article 5.3 guidelines ^19^ ^,^ ^20^ ^,^ ^21^. Consequently, the industry has publicized corporate social responsibility initiatives focused on reducing illicit trade and child labor ^19^, aligning its corporate activities with the United Nations Sustainable Development Goals ^21^, and engaging in corporate philanthropy ^20^. During the COVID-19 pandemic, tobacco companies increased their corporate social responsibility initiatives in Brazil, highlighting their efforts to provide supplies, funds, and COVID-19 test kits to hospitals and government health agencies, primarily in tobacco-growing areas ^16^.

Existing corporate social responsibility studies have primarily relied on document review methods to investigate strategies used by the tobacco industry, focusing mainly on marketing practices of a few specific companies. In contrast, this online experimental study adopted an innovative methodological approach to assess whether exposure to a fictitious tobacco company’s corporate social responsibility video affected Brazilian adults’ attitudes toward the company, support for tobacco control policies, and cigarette- and e-cigarette-related harm perceptions and intentions to use. This primary aim was informed by previous findings ^22^ ^,^ ^23^, suggesting that corporate social responsibility claims could affect outcomes by a “caring” mechanism, whereby a company’s socially responsible actions lead individuals to form more positive perceptions of its values and products ^24^. A secondary aim was to compare effects by gender and smoking status, based on evidence that women and men may respond differently to tobacco-related messaging ^25^ ^,^ ^26^ ^,^ ^27^ and that smoking status can shape attitudes and perceptions toward tobacco products ^28^. This study addressed methodological limitations and existing research gaps by providing experimental evidence on the effects of corporate social responsibility initiatives on specific constructs and population groups, going beyond prior research that has mainly explored companies’ motivations for publicizing their corporate social responsibility strategies.

## Materials and methods

### Eligibility, recruitment, and consent

Eligible participants were aged 18 or older and located in Brazil, as determined by IP address. Qualtrics (https://www.qualtrics.com/), an online survey provider, recruited participants in collaboration with Brazilian panel providers via email, in-app, and SMS invitations. The invitations provided general information about the study and participation incentives. To ensure adequate samples for subgroup analyses, we set “soft” quotas of 41-60% for past 30-day tobacco or nicotine (including e-cigarette) use (yes/no) and gender (men/women). We also set “soft” regional quotas to approximate Brazil’s population distribution: North (8%), Northeast (28%), Central-West (7%), Southeast (42%), and South (14%) ^29^. The University of Nevada, Reno (UNR) Institutional Review Board (IRB), and the Brazilian National Commission for Research Ethics determined that this study was exempt.

After confirming their age, participants reviewed a consent form broadly describing the study as “about tobacco marketing” and then completed sociodemographic questions. Participants were subsequently randomized (1:1) to view either a control or experimental video featuring a fictitious Brazilian tobacco company (“Cruzeiro do Sul”) created for this study. Participants answered questions regarding their: (a) attitudes toward Cruzeiro do Sul, (b) opinions about tobacco control policies, and (c) perceptions of the harm of the company’s cigarettes and e-cigarettes, as well as their intention to use these products. At the end of the survey, we informed participants that Cruzeiro do Sul was a fictitious company. We also disclosed the true purpose of the study and clarified that e-cigarettes cannot be legally produced or sold in Brazil. Data were collected between January and February 2023.

### Control and experimental videos

We designed the control ^30^ and experimental ^31^ videos to resemble actual tobacco company corporate social responsibility videos publicly available on YouTube and other social media platforms ^32^ ^,^ ^33^. The control video was three minutes long and highlighted the company’s business activities in Brazil (e.g., profits, products, and distribution locations). The experimental video was also three minutes long and contained the same information as the control video but also emphasized the company’s corporate social responsibility initiatives (e.g., support for family farming and local communities, environmentally friendly business practices, equity promotion, and racial inclusion). To equalize video length, the control video contained extra footage of tobacco farms and product distribution to compensate for the corporate social responsibility content included only in the experimental video. Both videos were originally filmed by a professional production company on the UNR campus, using nondescript office settings to mimic the interior of a Brazilian tobacco company. All actors were Brazilian graduate students and faculty members at UNR. See Supplementary Material (https://cadernos.ensp.fiocruz.br/static//arquivo/supl-e00111925_5972.pdf) for the experimental and control video scripts.

### Measures

The survey was developed in English, translated into Portuguese by a native Brazilian Portuguese speaker, and informally tested by 10 Brazilian adults to identify potential clarity issues. We used multiple items to assess attitudes toward Cruzeiro do Sul, support for tobacco control policies, and intention to use, modifying existing scales for each outcome. Perceived relative harm of cigarettes and e-cigarettes was measured using one item each. 

Attitudes toward Cruzeiro do Sul were measured using the following items: (1) “Cruzeiro do Sul is a socially responsible company”; (2) “I would like to see Cruzeiro do Sul go out of business”; (3) “Cruzeiro do Sul, like other tobacco companies, is hampered by the legislation in force in Brazil, compromising its profit and product supply”; (4) “Cruzeiro do Sul targets teenagers and young adults with its products and advertising”; (5) “Cruzeiro do Sul tells the truth about their products”; and, (6) “Cruzeiro do Sul does good things for local communities”. Responses were captured on a five-point Likert scale: “strongly agree”, “agree”, “neither agree nor disagree”, “disagree”, “strongly disagree”. Consistent with prior publications ^34^ ^,^ ^35^ ^,^ ^36^ ^,^ ^37^, we coded each response option so that higher values indicated better outcomes for public health. Items were adapted from the *2008 Adult California Tobacco Survey* and a *2021 Truth Initiative Survey*. We calculated a composite score by averaging responses across items, accounting for reverse-coded items. Scores could range from 1 (least favorable to public health) to 5 (most favorable). The initial six-item scale yielded a Cronbach’s alpha of 0.63 and an average inter-item correlation of 0.22 (range: 0.16-0.32). To improve internal consistency, items 3 and 4 were removed. The revised four-item scale achieved a Cronbach’s alpha of 0.76 and an average inter-item correlation of 0.44 (range: 0.40-0.52).

Support for tobacco control policies was assessed using the following items: (1) “I support a law banning smoking in outdoor public places”; (2) “Taxes on conventional cigarettes should not be increased”; (3) “I support a law raising the minimum age for purchasing conventional cigarettes from 18 to 21 years old”; (4) “I support a law prohibiting tobacco companies from sponsoring cultural, educational, and sporting events”; (5) “I support the government doing more to prevent companies from selling conventional cigarettes to people under 18”; (6) “I support the government doing more to prevent people under 18 from accessing electronic cigarettes”; (7) “I support the law that prohibits the manufacture and sale of electronic cigarettes in Brazil”; (8) “I support the current law requiring health warnings on cigarette packs covering 75% of the package”; and (9) “I support a law mandating standardized packaging for cigarette packs”. These items were adapted from the Second Brazilian National Survey on Alcohol and Drugs (BNADS). Response options followed the same five-point Likert scale and coding rationale previously above. The Cronbach’s alpha for the support of tobacco control policies was 0.81, with an average interitem correlation of 0.32 (range 0.31-0.35).

Relative perceived harm of Cruzeiro do Sul’s cigarettes and e-cigarettes was assessed with the following questions: (a) “Compared with other brands of conventional cigarettes on the market, how harmful do you think conventional cigarettes from Cruzeiro do Sul are to health?”; and (b) “Compared with other brands of electronic cigarettes already on the market (if manufacturing and sales were not prohibited), how harmful do you think electronic cigarettes from Cruzeiro do Sul are to health?”. Response options followed a five-point Likert scale: “a lot more harmful”, “more harmful”, “similarly harmful”, “less harmful”, “a lot less harmful”. We dichotomized outcomes by grouping “a lot more harmful” and “more harmful” as representing better outcomes for public health, and “similarly harmful”, “less harmful”, and “a lot less harmful” as representing poorer outcomes.

Intention to use cigarettes and e-cigarettes was assessed with the following questions: (a) “How likely are you to try a conventional cigarette made by Cruzeiro do Sul in the next six months?”; and (b) “How likely are you to try an electronic cigarette (if manufacturing and sales were not prohibited) made by Cruzeiro do Sul in the next six months?”. Response options followed a five-point Likert scale: “extremely unlikely”, “unlikely”, “neutral”, “likely”, and “extremely likely”. For interpretative consistency and alignment with public health priorities, responses were dichotomized into two categories: “extremely unlikely” and “unlikely”, representing better outcomes for public health (low intention to use), and “neutral”, “likely”, and “extremely likely”, representing poorer outcomes (greater intention to use).

Sociodemographic characteristics: Participants provided sociodemographic information (age, gender, race/ethnicity, income, educational level, and region) using standard items from the *Brazilian Census* ^38^.

Cigarette and e-cigarette use: following WHO’s classification of current users, which includes both daily and occasional use, we defined “current smokers” and “current e-cigarette users” as individuals who reported smoking/using e-cigarettes “every day” or “some days.” Individuals who reported smoking/using e-cigarettes “not at all” were classified as “nonsmokers” and “non-e-cigarette users.”

### Analyses

Qualtrics recruited a final sample of 4,047 participants after excluding responses based on the following criteria: (1) duplicate or non-Brazilian IP addresses (n = 6); (2) “straight-lining” behavior, defined as repeatedly selecting the same response option across matrix questions (n = 154); and (3) completion times shorter than two standard deviations below the median (n = 40).

We conducted multiple analyses to assess the effect of the experimental condition on key outcomes. Chi-squared tests were used to confirm that sociodemographic characteristics and smoking/e-cigarette variables were evenly distributed between control and experimental groups.

To evaluate the main effect of corporate social responsibility exposure on continuous outcomes - attitudes toward Cruzeiro do Sul and support for tobacco control policies - we applied linear regression models. We also included interaction terms (condition x gender and condition x smoking status) to assess whether effects differed across these subgroups. When interactions were significant, we conducted stratified analyses for more detailed interpretation.

For binary outcomes - perceived relative harm and intention to use cigarettes or e-cigarettes - we used Poisson regression models with robust variance estimation. This approach yields incidence rate ratios (IRRs), offering more intuitive interpretations than odds ratios. All analyses were conducted using Stata MP software (https://www.stata.com).

## Results

Table 1 presents characteristics of the study sample (n = 4,047), divided between the control group (n = 2,039) and the experimental group (n = 2,008). Participants were roughly evenly divided by gender (48.5% men, 51.5% women), and age was distributed across standard groups. Most participants identified as “White” (50%), followed by “Mixed-race” (36.2%) and “Black” (11.3%), reflecting national demographic patterns. Half the sample had completed an undergraduate degree (50.4%) and the majority resided in urban areas (95.2%). Monthly income exceeded BRL 5,001 (~USD 1,025.00) for approximately 38% of participants. Nearly 45% reported smoking cigarettes every or some days, and 27% reported using e-cigarette with the same frequency. All sociodemographic characteristics and cigarette/e-cigarette use statuses were evenly distributed by condition.

**Table 1:** Participant characteristics, overall and by experimental condition.

Parameters	Overall (N = 4,047)	Control condition (n = 2,039)	Experimental condition (n = 2,008)
%	%	%
Overall	100.0	50.4	49.6
Gender *			
Men	48.5	50.1	46.8
Women	51.5	49.8	53.1
Age (years)			
18-24	15.8	15.0	16.6
25-29	17.5	18.7	16.3
30-34	18.3	17.8	18.7
35-39	15.9	15.6	16.1
40-44	13.0	12.6	13.5
45-49	7.3	7.1	7.4
50+	12.2	13.2	11.2
Race			
White	50.0	51.2	48.8
Black	11.3	11.1	11.5
Yellow	1.9	1.5	2.4
Brown	36.2	35.6	36.7
Indigenous	0.4	0.3	0.4
Mixed-race	0.2	0.2	0.2
Education			
Incomplete middle school	1.5	1.7	1.3
Completed middle school	3.1	3.1	3.2
Incomplete high school	3.9	4.0	3.9
Completed high school	28.7	28.7	28.7
Incomplete undergraduate school	12.4	12.1	12.6
Completed undergraduate school	50.4	50.4	50.3
Zone			
Urban	95.2	95.2	95.1
Rural	4.8	4.8	4.9
Region			
North	9.9	9.9	9.9
Northeast	28.4	27.1	29.7
Central-West	8.6	8.2	9.1
Southeast	40.7	42.1	39.3
South	12.4	12.7	12.0
Income (BRL)			
1-500	3.9	3.9	3.8
501-1,000	6.7	7.1	6.3
1,001-2,000	15.3	15.1	15.5
2,001-3,000	15.7	16.4	15.1
3,001-5,000	20.4	19.4	21.4
5,001-10,000	23.0	23.5	22.5
10,001-20,000	10.2	9.8	10.5
20,001-100,000	3.7	3.6	3.8
˃ 100,001	1.1	1.1	1.1
Cigarette smoking status			
Nonsmokers	55.5	55.1	55.3
Smokes every day or some days	44.5	44.9	44.7
E-cigarette use status			
Non-users	73.0	72.9	72.9
Uses every day or some days	27.0	27.1	27.1

* One person was non-binary and was not included in the chi-squared analysis.

Supplementary Material (Table S1; https://cadernos.ensp.fiocruz.br/static//arquivo/supl-e00111925_5972.pdf) presents item-level results for attitudes toward Cruzeiro do Sul, support for tobacco control policies, relative perceived harm, and intention to use. Statistically significant differences by condition were observed for most items assessing attitudes toward the company, while no significant differences were found for items assessing support for tobacco control policies. Participants were more likely to perceive the company’s products as less harmful; however, differences in intention to try tobacco products were not statistically significant. Overall, the intervention appeared to influence attitudes toward the company and harm perceptions, without affecting policy support or intentions to use.

Table 2 presents regression results for attitudes toward Cruzeiro do Sul and support for tobacco control policies. Participants in the experimental condition reported significantly more positive attitudes than those in the control group (β = 0.16, 95%CI: 0.11; 0.21, p < 0.001). The interaction by smoking status was not significant (p = 0.20), whereas the interaction by gender was significant (p = 0.03). Stratified analyses revealed that the effect was nearly twice as large for women (β = 0.21, p < 0.001) as for men (β = 0.11, p < 0.01). No significant effect of the experimental condition was observed on support for tobacco control policies (β = -0.004, 95%CI: -0.043; 0.035, p = 0.88).

**Table 2:** Regression results for attitudes toward Cruzeiro do Sul and support for tobacco control policies.

Outcome	β	95%CI	p-value
Attitudes toward Cruzeiro do Sul	0.1604	0.1136; 0.2072	< 0.001
Support for tobacco control policies	-0.004	-0.0429; 0.0346	0.88

95%CI: 95% confidence interval.

Table 3 presents the results for relative perceived harm of Cruzeiro do Sul’s cigarettes and e-cigarettes, as well as intention to use these products. Participants exposed to the corporate social responsabilty video were 14% more likely to perceive cigarettes from Cruzeiro do Sul as less harmful (IRR = 1.14, 95%CI: 1.05; 1.25). The interaction by smoking status was significant (p = 0.04). Stratified results showed the association was significant for nonsmokers (IRR = 1.26, 95%CI: 1.04; 1.53) but not for smokers (IRR = 1.21, 95%CI: 0.99; 1.47). Gender also moderated the effect (p = 0.05), with a stronger and significant association for women (IRR = 1.21, 95%CI: 1.02-1.44) than for men (IRR = 1.14, 95%CI: 0.94; 1.38). No significant association was found between corporate social responsibility video exposure and perceived harm of e-cigarettes (IRR = 1.15, 95%CI: 0.94; 1.40), nor with intention to use cigarettes (IRR = 1.04, 95%CI: 0.86; 1.25) or e-cigarettes (IRR = 0.85, 95%CI: 0.71; 1.03) over the next six months. There was no evidence of moderation by smoking status or gender for these outcomes.

**Table 3:** Association between exposure to a corporate social responsibility message and relative perceived harm of the company’s products, as well as intention to use (N = 4,047).

	IRR (95%CI)	p-value
Relative perceived harm *		
“Compared with other brands of conventional cigarettes on the market, how harmful do you think conventional cigarettes from Cruzeiro do Sul are to health?”		
More harmful (reference) versus less harmful	1.14 (1.05; 1.25)	0.002
“Compared with other brands of electronic cigarettes already on the market (if manufacturing and sales were not prohibited), how harmful do you think electronic cigarettes from Cruzeiro do Sul are to health?”		
More harmful (reference) versus less harmful	1.15 (0.94; 1.40)	0.169
Intention to use **		
“How likely are you to try a conventional cigarette made by Cruzeiro do Sul in the next six months?”		
Unlikely (reference) versus likely	1.04 (0.86; 1.25)	0.681
“How likely are you to try an electronic cigarette (if manufacturing and sales were not to be prohibited) made by Cruzeiro do Sul in the next six months?”		
Unlikely (reference) versus likely	0.85 (0.71; 1.03)	0.087

95%CI: 95% confidence interval; IRR: incidence rate ratios.

* Construction of outcomes were “a lot more harmful”, “more harmful” versus “same harmfulness”, “less harmful” or “a lot less harmful”;

** Construction of outcomes were “neutral”, “unlikely” or “extremely unlikely” versus “extremely likely” or “likely”.

## Discussion

Our results demonstrate that viewing a corporate social responsibility video modeled on those widely circulated on YouTube and social media affected participants’ attitudes toward our fictional tobacco company and their perceptions about the relative harm of its cigarettes. Notably, the video we used was only three minutes long and contained a limited number of corporate social responsibility claims, substantially less content than typical real-world corporate social responsibility materials. Yet, it produced measurable shifts in attitudes and perceptions. Given the scope and frequency of tobacco companies’ corporate social responsibility campaigns in Brazil, real-world exposure - which is often longer, more persuasive, and repeated - may have an even greater effect on public attitudes toward companies, support for tobacco control policies, and perceptions of harm and intentions to use cigarettes and e-cigarettes ^16^ ^,^ ^19^ ^,^ ^20^ ^,^ ^21^.

Results suggesting that exposure to the corporate social responsibility video reduces perceived harm of our fictitious tobacco company’s cigarettes are consistent with the “health halo” effect, in which positive impressions created by attributes such as “organic” activate related concepts such as “healthy” which are then attributed to the product ^22^ ^,^ ^39^ ^,^ ^40^. Experimental studies have demonstrated health halo effects in product categories unrelated to tobacco, including snack foods ^41^, wine ^42^, and chocolate ^43^. In our study, exposure to the corporate social responsibility video highlighting Cruzeiro do Sul’s support for family farming and local communities, environmentally friendly practices, equity promotion, and racial inclusion may have influenced participants’ perception of the company as a caring and responsible entity. This may have extended to the company’s products, consistent with the belief that its cigarettes are less harmful ^24^.

Our study did not find evidence of a direct effect of corporate social responsibility initiatives on support for tobacco control policies. The lack of significant results may be due to item wording or incorrect assumptions about participants’ prior knowledge about tobacco control policies. Support for such policies may also be deeply held, making it less susceptible to brief exposure, as in this study. These findings should not be interpreted as evidence that real-world corporate social responsibility campaigns have no impact on policy support. Our brief corporate social responsibility claims presented here likely do not reflect Brazilians’ actual exposure to corporate social responsibility campaigns, which aim to transform often slow-to-change norms on a societal level ^44^.

Likewise, the absence of an effect of corporate social responsibility initiatives on intention to use the company’s products may reflect the limited information presented in the videos. Participants viewed a three-minute video about the company, with no detailed product information such as pricing, taste, visual aesthetics, or other attributes critical to consumers’ decisions to experiment a new tobacco product ^45^. These results align with previous literature, which generally shows that one-time exposure to messages rarely impacts intentions to use ^46^.

The WHO has highlighted the concerning influence of corporate social responsibility strategies on tobacco control policies and public perceptions of tobacco companies and their products ^47^ ^,^ ^48^. This concern stems from tobacco companies using corporate social responsibility to maintain visibility and engage with the public in the context of restrictions on traditional advertising. Consequently, corporate social responsibility initiatives function as a form of indirect advertising and sponsorship, replacing traditional direct marketing. The WHO FCTC Article 13 explicitly categorizes corporate social responsibility initiatives as a form of tobacco sponsorship, indicating the need for regulation ^49^. Furthermore, the guidelines for implementation of FCTC Article 5.3 ^49^ recommend denormalize, strictly regulate, or ban tobacco companies’ use of corporate social responsibility to prevent it from influencing public perceptions and hindering the development or implementation of public health policies ^49^. Our research underscores the importance of fully implementing the WHO FCTC and its Guidelines by adopting strategies to mitigate the impact of corporate social responsibility initiatives, such as explicitly categorizing corporate social responsibility as a form of tobacco sponsorship, banning it, or strictly regulating the advertisement and promotion of corporate social responsibility initiatives. The main goal is to ensure accurate information about tobacco companies’ business practices and products is disseminated to the public.

### Limitations

This study has several limitations. First, it used a fictitious tobacco company and simulated corporate social responsibility videos, which may not reflect the full complexity of real-world corporate social responsibility initiatives; however, this approach controlled for preexisting biases and ensured cultural relevance. Second, the sample - predominantly white, higher-income, and well-educated Brazilian adults - may not be representative of the general population. Lastly, since e-cigarettes are banned in Brazil, they were not depicted in the videos, which may have influenced participants’ responses regarding e-cigarette harm and intentions to use them.

## Conclusions

This study provided experimental evidence that including brief corporate social responsibility claims in a three-minute video significantly influenced viewers’ attitudes toward the tobacco company and their perceptions of the harm associated with its cigarettes. These findings underscore the deceptive impact of corporate social responsibility initiatives, which, like direct tobacco advertising, can undermine tobacco control efforts by enhancing the public image of tobacco companies. Our findings align with WHO FCTC recommendations and guidelines, supporting the need for clearer categorization and denormalization of corporate social responsibility initiatives, which could contribute to stronger tobacco control measures.
