# Supplementary material for: Association between tobacco company corporate social responsibility claims, support for the company and policies, and perceptions and intentions among Brazilians
Source: Cad Saude Publica. 2026 Feb 13;42:e00111925. doi: 10.1590/0102-311XEN111925 (PMC12931485; doi:10.1590/0102-311XEN111925)
Supplement: Supplementary Material [file 1678-4464-csp-42-EN111925-s.pdf]

Supplementary Material

Experimental Condition Script – Video 1

Scene 1 – Combination of CEO Voice Over and CEO speaking to camera:

Introduce the company
Opening – speaking to camera
"Nice to meet you! We are Cruzeiro do Sul Inc. Now I want to invite you to get to know our company"

Company's history in Brazil – speaking to camera
"Established in Brazil since 2019, Cruzeiro do Sul Inc. has been generating competitiveness through a fully integrated production chain that goes from the tobacco seed to the adult cigarette consumer. To secure the high standards, our company has two processing plants in the south of Brazil, guaranteeing the quality of the tobacco and its place as one of the largest tobacco exporters on the continent".

Company's finances – voice over
"Though we are a new company, Cruzeiro do Sul Inc. controls nearly 12 percent of the total tobacco market, while worldwide the company sells almost 62 billion cigarettes through 20,000 sales outlets."

Scene 2 – Scientists speaking to camera:

Introduce the products
Cigarettes produced and sold in Brazil - speaking to camera
"Our products undergo strict control at all stages of production, from growing tobacco to distributing cigarettes. Cruzeiro do Sul Inc. has access to the most modern tobacco technologies in Brazil, where we work to develop the highest quality, most satisfying cigarettes for Brazilian smokers."

Products - speaking to camera
"Our brands meet the demands of all consumer profiles. They are distinguished from each other by the mixture of types and classes of tobacco, in different proportions, giving each product its own flavors and aromas".

Scene 3 – Combination of CEO and Farmer Voice Over and CEO speaking to camera:

Introduce the company's social impact
Support for our farmers/CEO – speaking to camera
"Cruzeiro do Sul Inc has always seen family farming as the main pillar of our business. Cruzeiro do Sul Inc. supports our five thousand family farms with training on how to maximize profits, protect the earth, and make sure these family farms stay in the family for years to come. We also support initiatives to improve infrastructure and housing in rural areas in Brazil".

Personal testimonial/farmer - voice-over
"Becoming a Cruzeiro do Sul Inc employee changed my life and my family's life. Learning business skills and receiving the incentives to keep our family farm going has given us the financial stability we always wanted. Thank you, Cruzeiro do Sul Inc."

Sustainable business/CEO – speaking to camera
"Our concern for sustainability is not just in the field, but in all of the company's activities, ranging

from the choice of renewable energy matrices for factories, to encouraging good environmental practices by its employees".

Corporate conduct/CEO – speaking to camera + voice-over
"We believe in of the highest standards of ethics and transparency in all our activities. We want to be recognized as a responsible company in a controversial industry. But more than simply being a responsible company, we are also working towards earning public recognition for our responsibility. The responsibility of being a company that is deeply concerned with people's values, that has faith in the potential of each individual and believes that everyone should contribute. Following this line of thinking, we have implemented our "inclusive internship program" in which we focus on hiring not only black women but also young adults who have finished their education in public school. Contributing to social and racial justice is a priority to our company!"

Scene 4 – CEO speaking to camera:

Marketing Final Message - speaking to camera
"We stand out with the attitude to keep making a difference."
Nice to meet you! We are Cruzeiro do Sul Inc."

Control Condition Script – Video 2

Scene 1 – Combination of CEO Voice Over and CEO speaking to camera:

Introduce the company
Opening – speaking to camera
"Nice to meet you! We are Cruzeiro do Sul Inc. Now I want to invite you to get to know our company"

Company's history in Brazil – speaking to camera
"Established in Brazil since 2019, Cruzeiro do Sul Inc. has been generating competitiveness through a fully integrated production chain that goes from the tobacco seed to the adult cigarette consumer. To secure the high standards, our company has two processing plants in the south of Brazil, guaranteeing the quality of the tobacco and its place as one of the largest tobacco exporters on the continent".

"Our products are present in 90% of Brazilian cities, meeting a demand of approximately 10 thousand points of sale. Currently, Cruzeiro do Sul employs approximately 5,000 Brazilians"– *excerpt taken from the experimental video to reduce the time of the video.*

Company's finances – voice over
"Though we are a new company, Cruzeiro do Sul Inc. controls nearly 12 percent of the total tobacco market, while worldwide the company sells almost 62 billion cigarettes through 20,000 sales outlets."

"In Brazil, Cruzeiro do Sul's sales exceeded 1 billion reais in 2019, which made us one of the 5 largest corporations in the country"– *excerpt taken from the experimental video to reduce the time of the video.*

Scene 2 – Scientists speaking to camera:

Introduce the products
Cigarettes produced and sold in Brazil - speaking to camera
"Our products undergo strict control at all stages of production, from growing tobacco to distributing cigarettes. Cruzeiro do Sul Inc. has access to the most modern tobacco technologies in Brazil, where we work to develop the highest quality, most satisfying cigarettes for Brazilian smokers."

Products - speaking to camera
"Our brands meet the demands of all consumer profiles. They are distinguished from each other by the mixture of types and classes of tobacco, in different proportions, giving each product its own flavors and aromas".
"Considered one of the best in the world, the tobacco we use is exclusively supplied by our integrated rural producers" *– excerpt taken from the experimental video to reduce the time of the video.*

NO SCENE 3 FOR CONTROL VIDEO

Scene 3 completely excluded from the control video to fulfill research objectives.

Scene 4 – CEO speaking to camera:

Marketing Final Message - speaking to camera
"We stand out with the attitude to keep making a difference."
Nice to meet you! We are Cruzeiro do Sul Inc."

Table S1 Individual outcome items by experimental condition for attitudes about the company Cruzeiro do Sul, support for tobacco control policies, relative perceived harm, and intention to use.

	Control condition (n = 2,039)	Experimental condition (n = 2,008)	p-value *
	%	%	
**Attitudes about the company Cruzeiro do Sul**			
Cruzeiro do Sul is a socially responsible company			< 0.001
Strongly agree	53.9	62.3	
Agree	26.8	24.7	
Neither agree nor disagree	12.8	8.2	
Disagree	3.9	2.9	
Strongly disagree	2.7	1.9	
I would like to see Cruzeiro do Sul go out of business.			0.002
Strongly agree	8.2	6.8	
Agree	7.1	6.4	
Neither agree nor disagree	21.4	21.4	
Disagree	16.4	13.1	
Strongly disagree	46.9	52.3	
Cruzeiro do Sul, like other tobacco companies, is hampered by the legislation in force in Brazil, compromising its profit and the supply of products **			0.341
Strongly agree	16.7	17.2	
Agree	28.0	27.8	
Neither agree nor disagree	30.1	32.2	
Disagree	13.3	11.6	
Strongly disagree	12.0	11.3	
Cruzeiro do Sul targets teenagers and young adults with its products and advertising **			0.039
Strongly agree	20.8	22.4	
Agree	24.3	27.2	
Neither agree nor disagree	22.1	21.8	
Disagree	16.6	14.3	
Strongly disagree	16.2	14.5	
Cruzeiro do Sul tells the truth about their products			0.052
Strongly agree	57.2	60.6	
Agree	26.2	25.8	
Neither agree nor disagree	9.3	7.7	
Disagree	4.5	4.0	
Strongly disagree	2.9	1.9	
Cruzeiro do Sul does good things for the local communities			< 0.001
Strongly agree	39.5	51.0	
Agree	28.0	28.7	
Neither agree nor disagree	19.7	13.4	
Disagree	8.0	4.0	
Strongly disagree	4.8	3.0	
**Support for tobacco control policies**			
I support a law banning smoking in outdoor public places			0.787
Strongly agree	31.1	31.9	
Agree	16.9	16.4	
Neither agree nor disagree	15.7	15.7	
Disagree	16.9	15.7	
Strongly disagree	19.5	20.4	
Taxes on conventional cigarettes should not be increased			0.276
Strongly agree	27.0	27.9	
Agree	20.4	20.3	
Neither agree nor disagree	25.0	24.6	
Disagree	14.5	12.6	
Strongly disagree	12.0	14.7	
I support a law that raises the minimum age for buying conventional cigarettes from 18 to 21 years old			0.096
Strongly agree	46.6	48.5	
Agree	18.7	16.5	
Neither agree nor disagree	13.6	15.3	
Disagree	8.8	7.5	
Strongly disagree	12.3	12.3	
I support a law prohibiting tobacco companies from sponsoring cultural, educational, and sporting events			0.528
Strongly agree	33.5	34.3	

Agree	17.1	15.5	
Neither agree nor disagree	20.6	20.1	
Disagree	11.8	11.6	
Strongly disagree	17.1	18.5	
I support that the government should do more to prevent companies from selling conventional cigarettes to people under 18			0.761
Strongly agree	63.6	63.2	
Agree	15.1	15.2	
Neither agree nor disagree	10.8	11.8	
Disagree	4.9	4.3	
Strongly disagree	5.6	5.5	
I support that the government should do more to prevent people under 18 from accessing electronic cigarettes			0.346
Strongly agree	66.2	64.3	
Agree	13.6	14.7	
Neither agree nor disagree	10.73	11.9	
Disagree	4.4	4.9	
Strongly disagree	5.0	4.3	
I support the law that prohibits the manufacture and sale of electronic cigarettes in Brazil			0.659
Strongly agree	27.8	27.7	
Agree	14.5	13.3	
Neither agree nor disagree	22.2	23.4	
Disagree	14.6	15.4	
Strongly disagree	20.9	20.3	
I support the current law that mandates the inclusion of health warnings on cigarette packs, covering 75% of the package			0.587
Strongly agree	41.9	43.7	
Agree	22.8	20.8	
Neither agree nor disagree	21.8	22.3	
Disagree	7.9	7.8	
Strongly disagree	5.5	5.4	
I support a law that mandates the adoption of standardized packaging for cigarette packs			0.256
Strongly agree	27.1	27.7	
Agree	21.7	21.9	
Neither agree nor disagree	28.6	29.3	
Disagree	10.1	10.7	
Strongly disagree	12.6	10.3	
**Relative perceived harm**			
If we compare conventional cigarettes from Cruzeiro do Sul with other brands of conventional cigarettes that are on the market, how harmful do you think conventional cigarettes from Cruzeiro do Sul are to a person's health?			0.017
A lot more harmful	4.8	5.4	
More harmful	5.1	4.4	
Similarly harmful	58.3	53.8	
Less harmful	21.6	24.2	
Much less harmful	10.2	12.2	
If we compare electronic cigarettes from Cruzeiro do Sul (if manufacturing/sales were not to be prohibited) with other brands of electronic cigarettes that are already on the market, how harmful do you think electronic cigarettes from Cruzeiro do Sul are to a person's health?			0.006
A lot more harmful	4.8	6.9	
More harmful	7.4	6.7	
Similarly harmful	55.9	51.8	
Less harmful	22.4	23.9	
Much less harmful	9.6	10.7	
**Intention to use**			
How likely are you to try conventional cigarettes made by Cruzeiro do Sul in the next 6 months?			0.874
Extremely unlikely	31.9	30.6	
Unlikely	14.0	14.7	
Neutral	14.9	14.7	
Likely	22.9	23.0	
Extremely likely	16.4	17.1	
How likely are you to try electronic cigarettes made by			0.296

Cruzeiro do Sul in the next 6 months?		
Extremely unlikely	32.5	30.4
Unlikely	16.6	16.8
Neutral	14.6	16.8
Likely	21.9	21.6
Extremely likely	14.5	14.4

* p-values are from a chi square test;

** Item excluded from the final attitudes towards Cruzeiro do Sul scale.
